# A plasmon-electron addressable and CMOS compatible random access memory

**DOI:** 10.1126/sciadv.adr1172

**Published:** 2025-05-09

**Authors:** Shawn R. Greig, Curtis J. Firby, Triratna Muneshwar, Serhat Alagöz, Eric Hopmann, Brett N. Carnio, Mingyuan Zhang, Grace Ciarniello, Kenneth Cadien, Abdulhakem Y. Elezzabi

**Affiliations:** ^1^ Department of Electrical and Computer Engineering, University of Alberta, Edmonton T6G 2V4, Canada.; ^2^ Department of Metallurgical Engineering and Materials Science, Indian Institute of Technology, Bombay 400-076, India.; ^3^ Department of Physics, University of Alberta, Edmonton T6G 2E1, Canada.; ^4^ Department of Chemical and Materials Engineering, University of Alberta, Edmonton, Alberta T6G 2V4, Canada.

## Abstract

The rapid increase in data generation and storage poses substantial challenges, necessitating a transition from traditional charge-based devices to high-speed optical alternatives for computational tasks. Photon-assisted or plasmon-assisted memory devices emerge as promising solutions for facilitating faster read/write operations. By using surface plasmon polaritons for writing operations, we can dynamically read memory states through the measurement of tunneling currents in thin layers of HfO_2_ ferroelectric materials sandwiched between Au thin film electrodes. Our plasmon-addressable memory platform offers versatile functionality in both nanoelectronic and nanoplasmonic systems, demonstrating a robust hybrid architecture with transformative potential for computing and data processing applications.

## Teaser

A prototype of a bistable plasmonic-electronic RAM is presented using a ferroelectric tunnel junction.

## INTRODUCTION

The challenge of managing data has grown considerably in both economic and social contexts. Optimizing data processing is therefore of paramount importance. It is estimated that the required capacity of global data storage will reach 200 zettabytes (200 billion terabytes) by 2025, mainly driven by the rise of the Internet of Things, artificial intelligence, cloud-based computing, and big data analytics. The processing speed of this enormous volume of data has become the most critical bottleneck in computer systems. Over the past decade, there has been a transformative development in nonvolatile memory (NVM), revolutionizing the way data is stored and accessed (*1*–*3*). Unlike volatile random access memory (RAM), which temporarily stores data and gets wiped when the computer is turned off (*4*), NVM retains stored data even without the need for electrical power, thus enabling the electronic devices to preserve information persistently. NVM serves as the main building block in many devices, facilitating long-term data storage and empowering various applications including energy efficient neural networks for artificial intelligence (*5*). As the demand for versatile on-chip memory elements continues to grow, NVM’s role as a reliable and efficient solution remains indispensable in the evolving landscape of modern-day technology.

The quest for the optimal memory solution for integrated technology continues, fueled by the ever-growing demand for advanced memory systems compatible with established complementary metal-oxide semiconductor (CMOS) fabrication processes. However, the pursuit encounters substantial challenges, including incompatibility with existing microelectronic industry standards. Although all-electrically addressable memory schemes have shown promise for diverse data processing applications, the complexity of computational circuit networks presents additional hurdles. Slow access speeds, thermal-induced fault tolerance, noninterfering parallel reading/writing, and low power efficiency emerge as critical issues. These paramount concerns arise from the manipulation of fundamental charges, specifically electrons, through electrical means.

Transitioning away from charge-based devices to high-speed devices for computations and ultrafast artificial neural networks requires the utilization of either photons or plasmons (*6*–*11*). With recent advances in the integration of plasmonics and electronic platforms, an innovative pathway for future data storage must bridge the gap between electrons and plasmons to enable the creation of a single hybrid memory architecture with unparalleled capabilities. Up to now, basic plasmonic memory approaches, relying on modulating light intensity within waveguides, have been reported. These are achieved by electrically inducing nanoscale metal filaments within the light’s path (*12*) or via leveraging insulator-to-metal phase transition to modulate plasmon coupling (*13*, *14*). In these approaches, the memory state is written by an electrical signal, whereas the reading process relies upon the transmitted light detection. However, the integration of electron-photon interfaces on a single chip poses challenges due to the need for external optical-to-electrical signal conversion for the memory element (i.e., requiring a photon detector for each memory element). To overcome these limitations and enhance versatility, a more promising strategy would involve the utilization of a hybrid memory element that is both plasmonically addressable and electrically readable.

A paradigm shift in memory technology has transpired through the introduction of ferroelectric random access memory (Fe-RAM) and ferroelectric tunnel junctions (FTJs) (*15*–*17*). Ferroelectric (FE)–based memory devices combine the advantages of both dynamic RAM and flash memory, offering fast read and write speeds, low power consumption, high endurance, and nonvolatility. Typical FE memory devices consist of an FE layer sandwiched between two electrodes. As memory, they operate by using the unique polarization of noncentrosymmetric FE materials, which can retain a stable polarization state along one crystalline axis even in the absence of an electric field. The polarization state represents the stored data, with each polarization direction corresponding to a binary value (0 or 1). By applying an electric field, the polarization can be switched, allowing data to be written into FE memory cells and read as electrical current. An Fe-RAM couples this FE element with a field-effect transistor (FET) to act as a bistable capacitor, whose memory state is written by applying a voltage signal and is read as a current. Similarly, an FTJ acts as a voltage-controlled bistable resistor where the FE polarization state controls the flow of current. With high endurance levels (i.e., the ability of the device to withstand a certain number of read/write switching cycles), FE memory’s performance surpasses that of flash memory endurance, thus making it suitable for applications that require frequent data updates or intensive read/write operations. Fundamentally, at the basic structural level, an FTJ element is very similar to a typical nanoplasmonic metal-insulator-metal (MIM) waveguide (*18*–*20*). Therefore, a precisely tailored FTJ element can facilitate the propagation of surface plasmon polaritons (SPPs) wherein electrons absorb plasmons to overcome the potential barrier, allowing for electrical reading.

## RESULTS

Here, we present a prototype of a plasmonic-electronic RAM based on an FTJ. The incorporation of a few-nanometer-thick HfO_2_ as an FE material sandwiched between Au electrodes enables the tunnel junction to have two bistable states. These memory states can be effectively both addressed via an SPP signal and electrically read as a tunnel current. With this platform, we experimentally demonstrate dynamic reading and writing with data retention. Our investigation includes comprehensive analyses encompassing electrical, optical, material, and microscopy assessments, elucidating the underlying mechanisms of the nature of the SPP driven bistability and the origin of the memory retention phenomenon. Leveraging the inherent structure resembling a conventional FTJ tunnel memory, our plasmon-addressable FTJ-RAM (PFTJ-RAM) platform enables dual operation in both nanoelectronic and nanoplasmonic systems, highlighting a powerful hybrid architecture and offering transformative prospects for the landscape of computing and data processing.

**Fig. 1.**
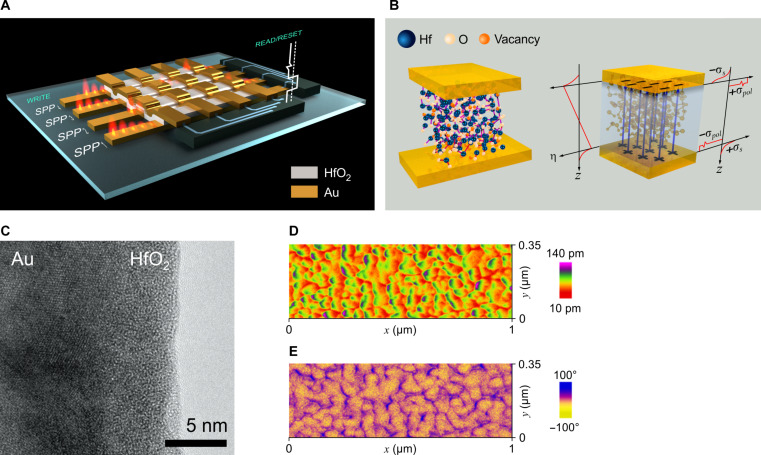
Experimental geometry schematics, transmission electron microscope images, and PFM images. (**A**) Schematic of the experimental geometry of a 16-cell element PFTJ-RAM array showing SPP writing and electrical reading arrangements. Image credit: W. Chang. (**B**) Left: An illustration of a single Au:HfO_2_:Au PFTJ-RAM element having Au electrodes and an oxygen vacancy-rich FE HfO_2_ insulating layer. Right: The Au-HfO_2_-Au junction showing Au electrode surface charge density distributions, σ*_s_*, HfO_2_ polarization charge density distributions, σ*_pol_*, and the respective electrostatic potential drop profile, η. The blue arrows indicate the polarization (P) of the HfO_2_ FE layer. (**C**) High-resolution bright-field transmission electron microscope image of the Au/HfO_2_ interface. Notably, the electron beam–evaporated Au film structure is polycrystalline whereas the 5-nm amorphous HfO_2_ film does not exhibit the typical FE orthorhombic crystal phase. The origin of the FE effect of HfO_2_ arises due to oxygen vacancies and the uncompensated polarization charges at the Au/HfO_2_ interfaces. (**D**) Piezoforce amplitude response from a 5-nm–thick HfO_2_ film on a 20-nm–thick Au electrode. (**E**) Piezoforce phase response from a 5-nm–thick HfO_2_ film on a 20-nm–thick Au electrode.

A schematic showing the SPP addressing and electrical reading of a 16-element Au:HfO_2_:Au PFTJ-RAM array is presented in Fig. 1A. To facilitate SPP coupling via the Kretschmann configuration, the device is mounted on a SiO_2_ prism. The structure consists of a thin film of oxygen vacancy-rich FE HfO_2_ (*21*–*27*), with a thickness of 3 to 5 nm, sandwiched between Au electrodes, forming a tunnel junction. To fully integrate the Au-based PFTJ-RAM in a monolithic CMOS device would require the use of a barrier layer composed of a material such as Ti, TiN, W, Ta, or TaN between the Au and underlying materials. This prevents the gold from diffusing into the silicon or metallic interconnects without negatively affecting device performance. Furthermore, the Au could also be replaced with another plasmonic metal such as Cu or Al without affecting the PFTJ-RAM behavior. Figure 1B provides a magnified cross-sectional view of a single Au:HfO_2_ interface, where the inherent polarization, P, of the FE HfO_2_ film (viz. the polarization surface charge density, σ*_pol_*) induces surface charge density, σ*_s_*, at the Au film, resulting in the creation of an inherent electrostatic potential η. This surface charge origin of the FE effect in the HfO_2_ layer is confirmed via the absence of the typical FE orthorhombic crystal phase when viewed in a transmission electron microscope (Fig. 1C) (see the Supplementary Materials for details). Shown in Fig. 1 (D and E) is the piezoforce microscopy (PFM) response from a 5-nm-thick HfO_2_ film on a 20-nm–thick Au electrode. The FE domains are evident where the amplitude response of the deflection signal (due to electric field–induced strain or deformation) is proportional to the local converse piezoelectric coefficient, and the phase specifies the local polarization orientation. Furthermore, the observation of a butterfly-like pattern in the amplitude and a hysteresis loop in the phase confirms that the HfO_2_ film is inherently piezoelectric and consequently exhibits FE behavior (see the Supplementary Materials for details).

**Fig. 2.**
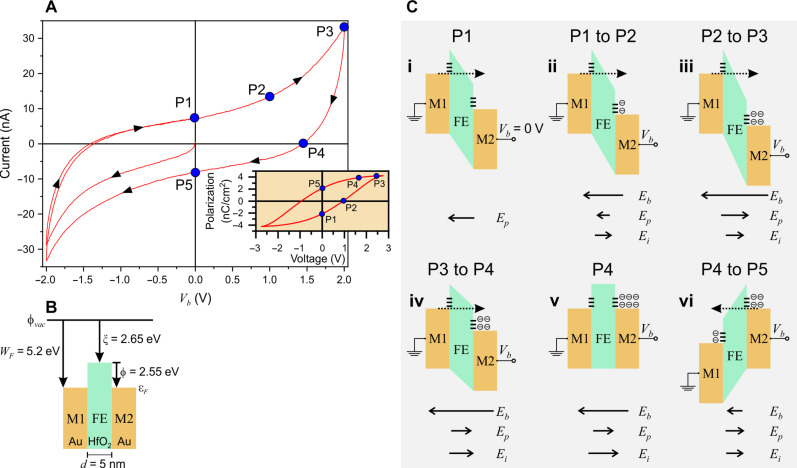
FE current-voltage characteristics and energy band of the PFTJ-RAM. (**A**) Current-voltage (*I*-*V*) curve of the PFTJ-RAM. P1, P2, P3, P4, and P5 are selected representative *I*-*V* points that are mapped onto the polarization-voltage curve (inset). Because of the *I*-*V* hysteresis, the curves are measured by initially cycling the device from 0 to −2 V and back to 0 V followed by cycling from 0 to +2 to −2 V and back to 0 V. The choice for the −2- to +2-V bias range ensures that the PFTJ-RAM is operated below its experimentally determined dielectric breakdown voltage of 0.71 V/nm. (**B**) Au:HfO_2_:Au PFTJ-RAM energy band diagram at zero bias voltage, *V_b_* = 0 V. *W_F_* (= 5.2 eV), work function of Au; ξ (= 2.65 eV), HfO_2_ electron affinity; ϕ, potential barrier height; ϕ*_vac_*, vacuum level energy; ε_F_, Fermi energy; and *d*, thickness of the HfO_2_ layer. (**C**) Energy band diagrams showing electron tunneling in the PFTJ-RAM with corresponding electric fields at the locations on the *I*-*V* curve presented in (A) at (i) P1, (ii) P1 to P2, (iii) P3, (iv) P3 to P4, (v) P4, and (vi) P4 to P5. The strength of the corresponding electric fields is represented by the lengths of the arrows.

To discern the electrical characteristics displayed by the FTJ, Fig. 2A depicts the current versus voltage (*I*-*V*) curve without SPP addressing of the memory element. Evidently, a noticeable current flow of 7 nA is observed in the absence of any applied bias across the memory cell at point P1. This characteristic is consistently observed across multiple devices, including those having a 3-nm HfO_2_ FE film, which additionally exhibit negative differential resistance (NDR) (see the Supplementary Materials for details). Upon applying a negative voltage of −*V_b_*, the *I*-*V* curve exhibits a hysteresis loop, which is attributed to the spontaneous electric polarization of the HfO_2_ layer. Notably, the device’s *I*-*V* hysteric behavior is the fundamental characteristic that is harnessed in the electrically activated Fe-RAM devices (*4*). This hysteresis arises due to a number of effects including the FE properties of the HfO_2_ insulating layer and the presence of oxygen vacancies and defect trap states at each of the Au-HfO_2_ interfaces. These vacancies are prevalent inherent defects that occur frequently in HfO_2_ thin films at a high concentration of ~1.7 × 10^21^ cm^−3^ (*21*). Under the influence of a high electric field or thermal excitation, the migration of oxygen vacancies from the interface results in the formation of the FE polar orthorhombic *Pca*2_1_ phase (*21*, *25*). The slight asymmetry between the positive and negative sides of the tunneling currents is attributed to the difference in the interface defect states at each interface as the order of the material deposition (i.e., HfO_2_ on Au versus Au on HfO_2_) dictates the local FE response of the material.

The observed hysteresis in both the *I*-*V* and the PFM measurements are a prelude to plasmon addressing of the FTJ-RAM cell. This analysis is guided by the behavior of the energy band diagram at points P1 to P5 on the *I*-*V* curve in Fig. 2A, with the energy band diagram at *V_b_* = 0 in Fig. 2B serving as a reference. The Au work function, *W_F_* (= 5.2 eV), and the HfO_2_ electron affinity, ξ (= 2.65 eV) (*28*), result in a potential barrier ϕ of 2.55 eV. When a bias voltage, *V_b_*, is applied to the PFTJ-RAM cell, three electric fields emerge: (i) *E_b_*, due to the externally applied *V_b_*; (ii) *E_i_*, arising from the tunneling electrons accumulating at the HfO_2_-Au interface states; and (iii) the FE polarization electric field, *E_p_*, inside the HfO_2_ film, which contributes for the depolarization electric field, *E_depol_*, that arises due to incomplete image charge compensation of the FE polarization-induced charges (*29*, *30*). It is the interplay between these electric fields that allows the PFTJ-RAM device to be either electrically and/or plasmonically addressed.

In a closed circuit, at P1 in Fig. 2A, and when *V_b_* = 0 (i.e., *E_b_* = 0), *E_p_* is nonzero due to the remnant polarization of the HfO_2_ film. Here, the Fermi level, ε*_F_*, is shifted, altering the shape of ϕ and electrons at the tail of the Fermi distribution tunnel from M1 to M2 to compensate for the potential change and a steady state is achieved, as illustrated in Fig. 2C(i). When *V_b_* is increased along the path from P1 to P2, *E_b_* increases whereas *E_p_* decreases as more image charges are compensated, resulting in the rise of the number of electrons tunneling across the potential barrier, as depicted in Fig. 2C(ii). However, a large fraction of these electrons accumulate at the FE-M2 interface states (i.e., oxygen vacancies and defect trap states) resulting in the formation of an internal field *E_i_*. At P2 (*V_b_* = 1 V), the FE polarization reverses direction, and as *V_b_* increases along the path from P2 to P3, *E_p_* increases following the FE polarization curve (Fig. 2A, inset) but in opposition to *E_b_*. Because *E_b_* > (*E_p_* + *E_i_*), more electrons tunnel from M1 to M2 as shown in Fig. 2C(iii), thus increasing the tunneling current. Beyond *V_b_* = 2 V (*E_b_* = 0.4 V/nm), noticeable evidence of trap accumulation would manifest as an NDR in the *I*-*V* curve when the FE layer thickness is ≤3 nm (fig. S5). However, at this bias voltage, *E_b_* approaches the experimentally determined dielectric breakdown voltage of 0.71 V/nm. When the voltage *V_b_* is decreased from P3 to P4, the current does not retrace its original trajectory; instead, it experiences a steep decline. This is because the interface states, which are only partially populated at P3, continue to be filled with electrons [Fig. 2C(iv)] as the current flows from M1 to M2 [i.e., *E_b_* > (*E_p_* + *E_i_*)]. This, in turn, increases *E_i_*; meanwhile, *E_p_* reduces (i.e., P is less at lower *V_b_*). At P4, the population of trapped electrons is large enough such that (*E_i_* + *E_p_*) = *E_b_*, resulting in zero net effective field, and thus, ε*_F_* shifts back to its flatband position [Fig. 2C(v)]. The counterbalancing of the electric fields results in no electrons tunneling between the contacts. As *V_b_* further decreases from P4 to P5, *E_i_* and *E_p_* now dominate where (*E_i_* + *E_p_*) > *E_b_* and electrons flow in the opposite direction from M2 to M1, reversing the current flow (i.e., negative current) and emptying the trapped electrons, as depicted in Fig. 2C(vi). For negative *V_b_*, the behavior is similar but with reversed electric field directions.

**Fig. 3.**
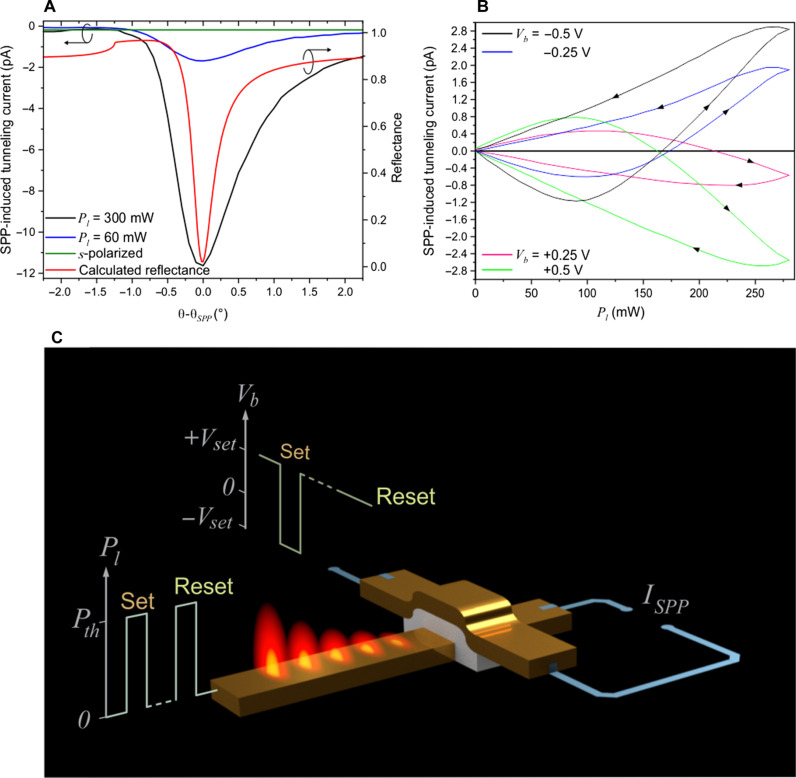
SPP-induced tunneling current and Set/Reset operation. (**A**) SPP-induced tunneling current dependence on laser light incidence angle for *p*-polarized incident laser light, *s*-polarized incident laser light, and the calculated theoretical reflectance from the Au-HfO_2_-Au thin film stack. (**B**) SPP-induced tunneling current as a function of incident laser power for different bias voltages. The unbalanced hot electron distribution between M1 and M2 appears as a slight asymmetry between the *V_b_* > 0 and *V_b_* < 0 curves. (**C**) Set/Reset operations of the PFTJ-RAM. For the Set operation, the memory cell is cycled from *V_set_* = +0.5 V to *V_set_* = −0.5 V via a bipolar electrical pulse and at the same time applying *P_l_* > *P_th_*. For the Reset operation, the memory cell is subjected to *P_l_* > *P_th_* with *V_set_* = 0.5 V.

Although we have shown that electrical manipulation can alter elements such as tunnel barrier height, electron trapping, and current flow direction, we will also demonstrate how this memory cell’s control can extend to SPP excitation using an 800-nm wavelength laser light at various power levels, *P_l_*. As the Au-HfO_2_-Au PFTJ-RAM structure matches typical MIM plasmonic waveguides (*20*), SPPs are excited on the electrically biased PFTJ-RAM and the subsequent SPP-induced tunneling current is measured through lock-in detection (see fig. S6 for the plasmonic mode profile). The SPP field excites electrons in the Au films above the Fermi energy level, ε*_F_*, creating so-called hot electrons (*31*), or excites electrons from negatively charged oxygen **V**^−^ and **V**^2−^ vacancy centers located at 1.24 and 0.99 eV below the conduction band of the HfO_2_ film, respectively (*32*). Our findings reveal a complete absence of plasmon-drag current (see the Supplementary Materials for details). Figure 3A depicts the measured tunneling current as a function of the incidence angle and the theoretically calculated reflection curve for the Au-HfO_2_-Au thin film stack (see fig. S8 for full schematic of the illumination). The observation that tunneling current is not present for *s*-polarization, combined with the finding that the highest current occurs for *p*-polarization at the SPP coupling angle, θ*_SPP_*, indicates that the origin of tunneling current is a result of the SPP excitation.

Figure 3B shows the measured tunnel current induced by the SPP, *I_SPP_*, in a single PFTJ-RAM element, within a voltage range of *V_b_* = −0.5 V to +0.5 V (see fig. S8 for a higher granularity of *V_b_* curves.) The set of curves shows similarity at a given absolute value of *V_b_*, with the direction of electron flow determined by the polarity of *V_b_*. At any *V_b_* value, the SPP-induced tunneling current exhibits two states characterized by the opposing directions of electron flow (i.e., from M1 to M2 or M2 to M1), which is akin to that observed in the electrically addressed FTJ in Fig. 2A. The slight asymmetry between the curves for *V_b_* > 0 and *V_b_* < 0 illustrates the uneven hot electron distribution between M1 and M2.

The most notable showcasing of the PFTJ-RAM cell is that, at a specific *V_b_* value, there exists a critical threshold of the incident laser power, *P_th_* (ranging from 150 to 220 mW), where the phenomenon of SPP-induced tunnel current completely ceases despite the presence of the externally driving electric field, *E_b_*. This behavior is similar to what was observed previously when the PFTJ-RAM was electrically addressed at *V_b_* = 1.5 V [i.e., P4 in Fig. 2A, where the population of trapped electrons becomes large enough such that (*E_i_* + *E_p_*) = *E_b_*, resulting in zero net effective field and shifting ε*_F_* back to its flatband position]. Even more intriguing is that, when the excitation laser power is reduced below *P_th_*, the SPP-induced tunneling current flows in the opposite direction from M2 to M1 akin to reversal of current flow as *V_b_* goes from P4 to P5 in Fig. 2A, where (*E_i_* + *E_p_*) > *E_b_* and the electrons are released from the oxygen vacancy states.

As the SPP-induced tunnel current direction in the PFTJ-RAM is bistable, it can be Set and Reset similar to a conventional FTJ. When operating at *V_b_* > 0, to Set the device, a negative voltage pulse of −*V_set_*, where *V_set_* = 0.5 V, is applied while simultaneously exciting SPP at *P_l_* > *P_th_*, whereas, to Reset the PFTJ-RAM, *V_b_* is kept at +*V_set_* while simultaneously exciting SPP at *P_l_* > *P_th_*, as depicted in Fig. 3C. Conversely, when operating at *V_b_* < 0, the role of +*V_set_* and −*V_set_* are simply reversed.

**Fig. 4.**
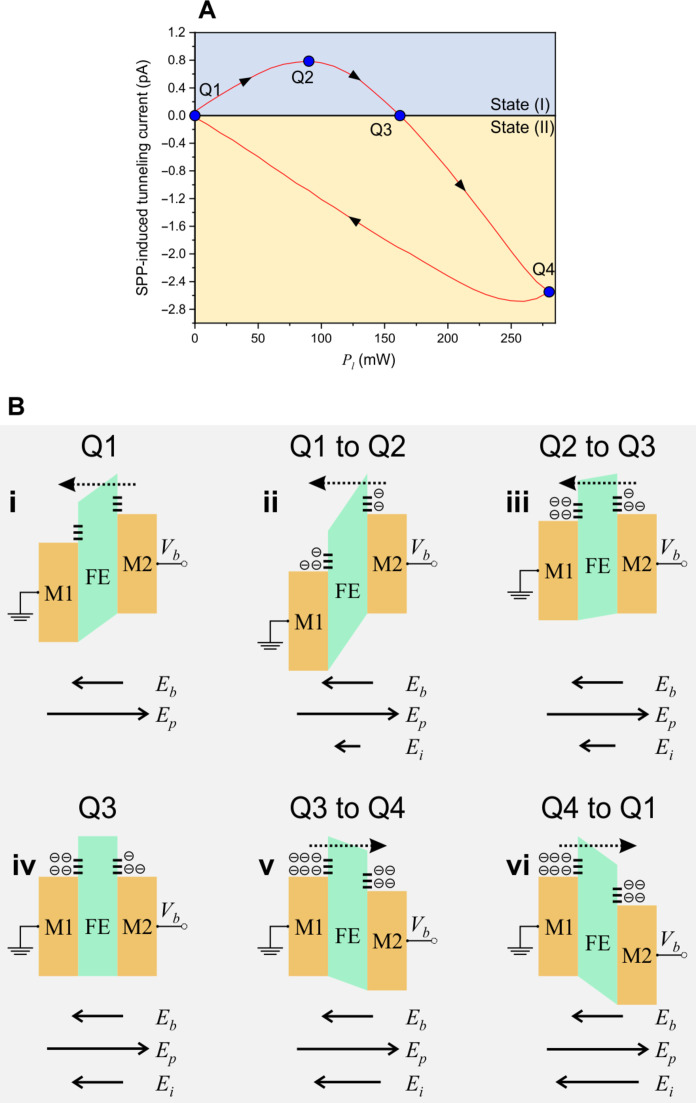
PFTJ-RAM SPP-induced tunneling current and band diagrams. (**A**) Exemplary SPP-induced tunneling current at *V_b_* = +0.5 V as a function of incident laser power. The Q1, Q2, Q3, and Q4 points represent the tunneling current flow direction at different effective electric fields. (**B**) Energy band diagrams illustrating the SPP-induced tunneling current. The energy band diagrams illustrate electron tunneling in the PFTJ-RAM with corresponding electric fields at specific points on the *I*-*P_l_* curve in (A). M1, M2, Au; FE, HfO_2_; *E_b_* is the externally applied field; *E_p_* is the FE polarization electric field; *E_i_* is the electric field arising from the tunneling electrons accumulating at the HfO_2_-Au interface states. (i) At Q1. (ii) Along Q1 to Q2. (iii) Along Q2 to Q3. (iv) At Q3. (v) Along Q3 to Q4. (vi) Along Q4 to Q1.

To clarify the origin of the bistability in the observed tunneling current, we analyze an exemplary curve at *V_b_* = +0.5 V (Fig. 4A) using the energy band diagrams depicted in Fig. 4B. The energy band diagram at Q1 is illustrated in Fig. 4B(i), with the electric field directions and relative magnitudes indicated by their represented arrows orientation and length, respectively. Here, because *E_p_* > *E_b_*, it is favorable for electrons to tunnel from M2 to M1, but because there is no laser excitation, no SPP-induced tunneling current is present. Progressing along the curve of the current induced by SPPs from Q1 to Q2, the power (i.e., number of SPP quanta) is increasing, thus increasing the population of hot electrons that tunnel from M2 to M1 because *E_p_* is still greater than *E_b_*. As the electrons tunnel from M2 to M1, oxygen vacancies and defect states at the FE-M1 interface start to fill. This population of electrons induces an internal field *E_i_*, similar to that in the electrical *I*-*V* measurement. This process is schematically depicted in Fig. 4B(ii).

As the laser power continues to increase from Q2 to Q3, the number of electrons in the FE-M1 traps increases such that *E_i_* increases and (*E_b_* + *E_i_*) ≈ *E_p_* [Fig. 4B(iii)]. Once Q3 is reached, there is no net electric field as (*E_b_* + *E_i_*) cancels out *E_p_* and the barrier will be in its flatband position, resulting in no current flow at this laser power, as shown in Fig. 4B(iv). Progressing along from Q3 to Q4, *E_i_* continues to increase due to having more hot electrons, but now (*E_b_* + *E_i_*) > *E_p_*, which causes the current to flow in the opposite direction (i.e., flow from M1 to M2, driven by *E_b_* and *E_i_*), as illustrated in Fig. 4B(v). As laser power is decreased along the path from Q4 to Q1, the traps become highly populated with electrons such that the net electric field (i.e., *E_b_* + *E_i_* – *E_p_*) is constant and the height and shape of the barrier does not change, as shown in Fig. 4B(vi). This manifests as a linear SPP-induced tunneling current (e.g., in the region where *P_l_* is between 250 and 0 mW). The slight nonlinearity in the SPP-induced tunneling current in the 280- to 250-mW region suggests that the traps are not completely filled with electrons at *P_l_* = 280 mW. The only change that occurs along the path from Q4 to Q1 is the overall number of electrons induced by SPPs consequently affecting the measured SPP-induced tunneling current.

**Fig. 5.**
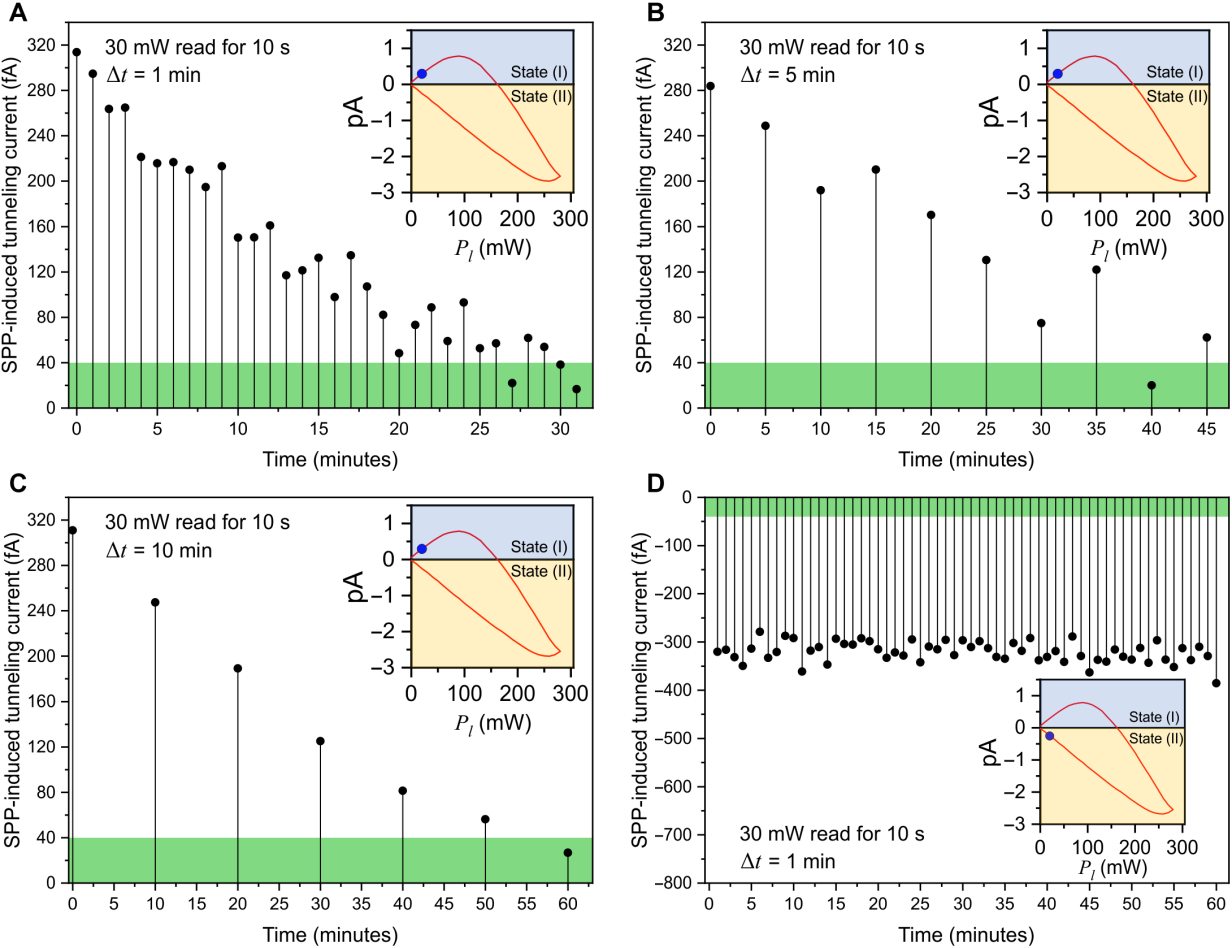
PFTJ-RAM retention during Set/Reset operations. Reading State (I) for 10 s every (**A**) 1, (**B**) 5, and (**C**) 10 min. The green colored band depicts the threshold level in reading the SPP-induced tunneling current as determined by the signal-to-noise ratio in the measurement system. In the Reading State (I), the data retention is >30 min. (**D**) Reading State (II) for 10 s every minute for >60 min. The insets illustrate specific points along the complete curve of the SPP-induced tunneling current being read.

Now, we evaluate the feasibility of the PFTJ-RAM as a functional memory element. In this context, the memory has two specific states: State (I), which corresponds to the flow of positive current, and State (II), which corresponds to the flow of negative current. The Read operation is performed with no voltage bias and at *P_l_* = 30 mW with the Set and Reset conditions described previously (Fig. 3C). Equally critical to performance is the PFTJ-RAM memory state retention because it determines the ability of a memory cell to store and maintain information over time. A reliable memory must be able to retain its stored states for long durations without considerable deterioration or loss. The memory State retention of the PFTJ-RAM was measured by performing a Set operation followed by an SPP-induced tunneling current Read operation for ten seconds with a specific time interval in between the successive Reads until the State is diminished. Figure 5 (A to C) depicts the readings of memory State (I) for three independent time intervals of 1, 5, and 10 min, respectively. The PFTJ-RAM demonstrates notable retention capabilities. Specifically, it maintains its state for 30 min when it is read at 1-min intervals. In addition, when read at 5-min intervals, it preserves its state for 45 min, and at 10-min intervals, it retains its state for 50 min. Similarly, memory State (II), where the PFTJ-RAM was Reset, and readings were taken every minute. It was determined that the memory State (II) represents a more stable memory state when being read every minute for more than 60 min without showing signs of degradation during or after this interval (Fig. 5D). The way traps are formed at the interface between the Au electrodes and HfO_2_ FE material is crucial. Although the junction itself is symmetric, the difference between State I and State II endurance is likely resulting from the response of the trap defect states at each interface (due to the order of deposition Au/HfO_2_ versus HfO_2_/Au) due to the polarity of the surface charge density distributions.

## DISCUSSION

Although the overall endurance of the PFTJ-RAM is not as high as that of a typical Fe-RAM, it is important to note that the PFTJ-RAM is still in its early stages of development as a rudimentary memory device. Time-resolved measurements of the current on the femtosecond timescale are crucial for unraveling the fundamental origin of the tunneling current as they can distinguish between ultrafast photo-assisted tunneling from plasmon-generated hot carriers and the slower responses associated with defect state–mediated processes. With improved control of defect states and further exploration of alternative FE materials and electrodes, the endurance of the PFTJ-RAM can be greatly enhanced, paving the way for even more reliable and robust memory performance. Furthermore, scalability and plasmonic losses present challenges, but they also open avenues for innovative engineering solutions and further research into the integration of optical and electronic systems.

Our experiments facilitate the dynamic writing and reading of memory states in CMOS-compatible FTJs. This is accomplished by harnessing SPP signals at the metal/dielectric interface for writing and tunnel current for reading memory states. The controls and characterization outlined in our study provide a crucial foundation for the development of memory devices capable of operating at higher speeds and improved energy efficiency.

## MATERIALS AND METHODS

### FE HfO_2_ atomic layer deposition

Hafnium oxide (HfO_2_) films were deposited using a continuous flow ALD-150 LX reactor manufactured by K. J. Lesker. The reactor used a total argon (Ar) flow rate of ~1000 standard cubic centimeters per minute (SCCM), consisting of 99.999% pure Ar supplied by Praxair. The resulting pressure inside the reactor was maintained at around 1 torr. Tetrakis(dimethylamido)hafnium (TDMAHf; 99.99% purity from Sigma-Aldrich) was used as the Hf precursor, whereas for oxygen supply, a remote inductively coupled O-plasma (ICP) operating at a frequency of 13.56 MHz and power of 0.6 kW was used. TDMAHf molecules were delivered from an ampoule to the reactor under 40-SCCM Ar carrier gas, where the precursor ampoule, ALD valve, and the delivery line were heated to 75°, 100°, and 110°C, respectively. In the O-plasma exposure step, a 60-SCCM flow of O_2_ (99.995%, Praxair) was introduced into 100-SCCM Ar carrier gas stream through ignited ICP confinement. The plasma-enhanced atomic layer deposition (PEALD) HfO_2_ films were grown at a 150°C substrate temperature with each deposition cycle consisting of 0.06-s TDMAHf pulse, 8-s purge, 5-s O-ICP exposure, and 5-s purge. To facilitate TDMAHf half-reaction on Au metal surface during HfO_2_ PEALD, substrates were subjected to 30-s O-ICP plasma exposure prior to HfO_2_ deposition (*33*).

### Electrode deposition

The bottom and top Au electrodes are deposited via electron-beam evaporation at a rate of ~1 Å/s and a base pressure of 1.2 × 10^−6^ torr.

### Measurement setup

Current-voltage (*I*-*V*) measurements were obtained using a calibrated Keithley 2400 in conjunction with a probe station. For all measurements, the bottom Au electrode, M1, is grounded and a bias voltage, *V_b_*, is applied to the top Au electrode, M2. In addition, independent measurements were carried out using a calibrated Keithley 4200. Both instruments generated *I*-*V* curves that were perfectly identical.

For the SPP experiments, the PFTJ-RAM sample was mounted on a fused silica prism to facilitate excitation of surface plasmons by coupling an 800-nm wavelength laser light from a Ti:Sapphire oscillator via the Kretschmann geometry. An angular translation stage was used to fine-tune the SPP coupling to achieve the highest SPP-induced current. The top and bottom electrodes are electrically connected to a lock-in amplifier (Stanford Research SR830) taking its reference from an optical chopper (Stanford Research SR540) placed in the laser beam path. The use of a lock-in amplifier allows the detection of only those electrons that were generated during SPP excitation.

## Supplementary Materials

### This PDF file includes:

Supplementary Text Figs. S1 to S10 References
