# Supplementary material for: A plasmon-electron addressable and CMOS compatible random access memory
Source: Sci Adv. 2025 May 9;11(19):eadr1172. doi: 10.1126/sciadv.adr1172 (PMC12063659; doi:10.1126/sciadv.adr1172)
Supplement: Supplementary file 1 — Supplementary Text Figs. S1 to S10 References [file sciadv.adr1172_sm.pdf]

Science Advances
AAAS

Supplementary Materials for

A plasmon-electron addressable and CMOS compatible random access memory

Shawn R. Greig *et al.*

Corresponding author: Shawn R. Greig, sgreig@ualberta.ca

Sci. Adv. **11**, eadr1172 (2025)
DOI: 10.1126/sciadv.adr1172

This PDF file includes:

Supplementary Text
Figs. S1 to S10
References

Supplementary Text

HfO_2 layer Thickness Characterization

The thickness of the PEALD HfO_2 films was determined using in-situ spectroscopic ellipsometry measurements carried out with a M2000DI instrument from J. A. Woollam. The measurements were performed in the spectral range of 0.73 – 6.4 eV. For the analysis of the ellipsometry data, the dielectric function of Au and HfO_2 were characterized using the Drude-Lorentz and Tauc-Lorentz parametrization methods, respectively. The growth per cycle (GPC) for the HfO2 PEALD process was determined by calculating the slope of the thickness versus deposition cycle. (33)

Piezoforce Microscopy

Piezoforce microscopy (PFM) of the PFTJ-RAM was carried out using a Bruker Dimension Fast Scan AFM (Santa Barbara, USA) in contact mode to confirm the ferroelectric behavior of the PFTJ-RAM. A CoCr coated tip (AppNano) was used to measure the piezo response of the PFTJ-RAM. The PFTJ-RAM was first poled by applying +2 V tip bias in contact mode while scanning a 1 µm × 0.35 µm area four times. In order to measure the piezo response, a +10 V tip bias was used at a low-speed piezo response (L-SPR) amplitude and frequency of +10 V and 15 kHz, respectively, at a scan rate of 0.5 Hz. The observation of the butterfly-like pattern (Fig. S1A) in the amplitude and the hysteresis loop in the phase (Fig. S1B) confirms that the HfO_2 is inherently piezoelectric and consequently exhibits ferroelectric behavior.

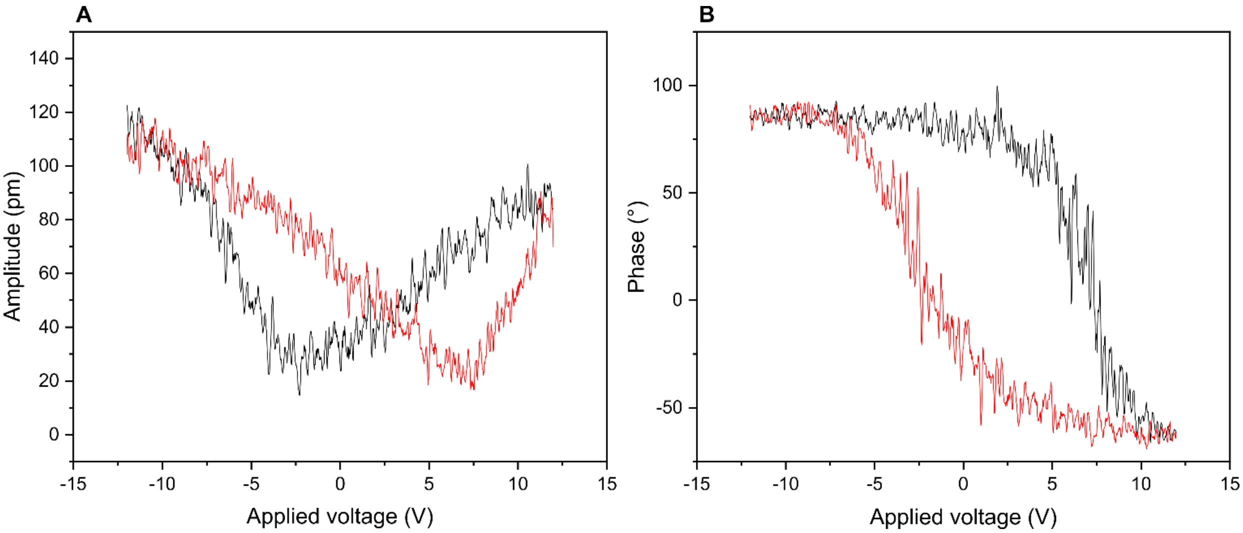

Fig. S1. Piezoforce microscope amplitude and phase. **(A)** Amplitude and (**B**) phase of the piezoforce microscopy (PFM) signal. The amplitude and the shape of the butterfly-like pattern are indicative of ferroelectric domain orientation and piezoelectric characteristics of the HfO_2 layer. The hysteresis in the phase signal is due to the orientation of the piezoelectric dipoles in the HfO_2 layer.

Transmission Electron Microscopy

Transmission electron microscope (TEM) measurements were performed using a JEOL JEM-ARM200CF S/TEM with specific attention being paid to the interface between the Au and the HfO_2 layers to confirm the origin of the ferroelectric behavior of the PFTJ. In order to facilitate the TEM measurements, the sample was prepared on a lacey carbon TEM grid (Ted Pella) with a

20 nm Au layer covered with a 5 nm HfO_2 layer. Fig. S2 depicts the elemental mapping images of O, Hf, and Au at the Au/HfO_2 interface.

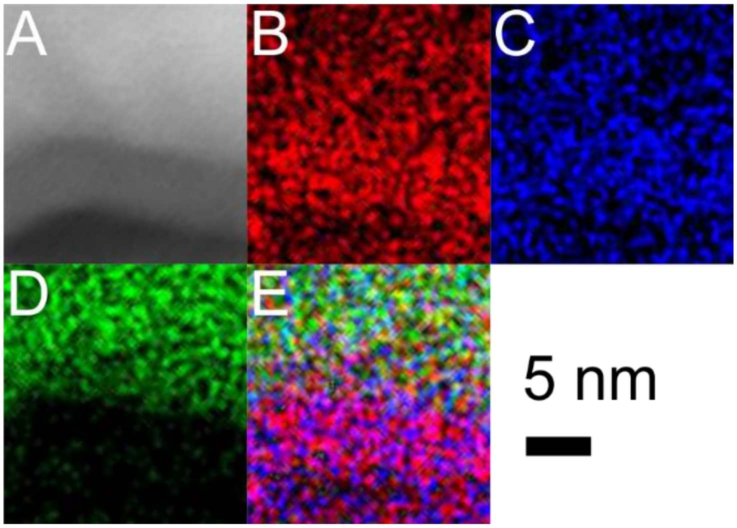

Fig. S2. **Transmission electron microscope images. (A)** Transmission electron microscope (TEM) images of the sample. Elemental mapping of: **(B)** Hf, **(C)** O, **(D)** Au, and **(E)** all of the elements combined.

Grazing-Incidence X-ray Diffraction

To verify the amorphous nature of the HfO2 layer, grazing-incidence X-ray diffraction (GI-XRD) was conducted on the PFTJ sample using a Rigaku Ultima IV XRD system. The measurements were performed with an incidence angle of 0.5°. Here, there is no top Au layer present in order to access the HfO_2 layer on top of the bottom Au layer. Fig. S3 illustrates the obtained XRD spectrum. The presence of broadened peaks at 20° and 30° degrees, coupled with the absence of prominent diffraction peaks at 28.5° and 31.5° (indicative of the monoclinic phase), as well as at 30.5° (associated with the orthorhombic and tetragonal phases), confirms the amorphous nature of the HfO_2 layer.

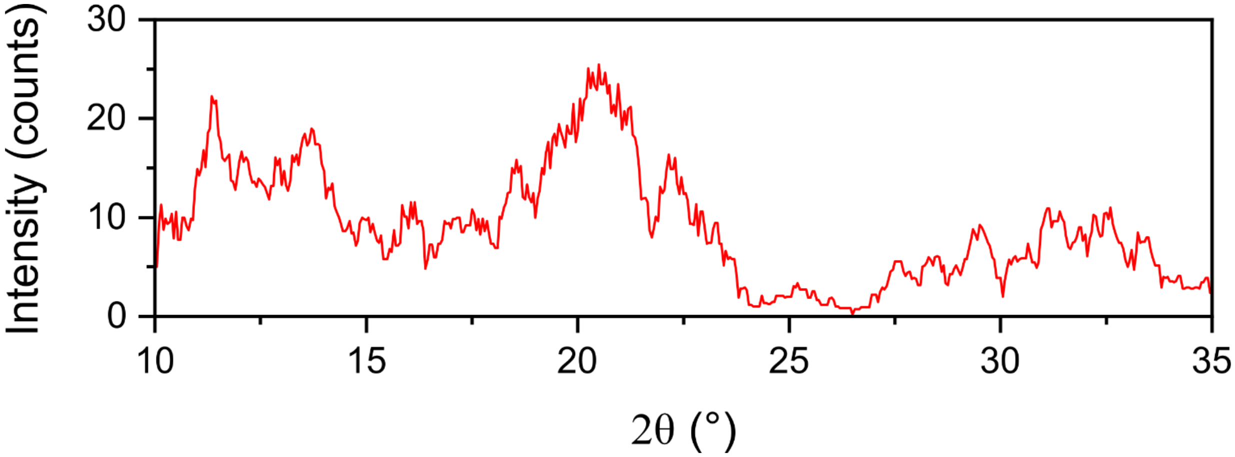

Fig. S3. **Grazing-incidence X-ray diffraction of the HfO_2 layer.**

X-ray Photoemission Spectroscopy

X-ray photoemission spectroscopy (XPS) measurements using a Kratos Axis XPS system were performed on the PFTJ-RAM sample to confirm the absence of carbon doping in the HfO_2 layer which might be responsible for the ferroelectric effect. Here, only the bottom Au and HfO_2 layers are present. Fig. S4 depicts the measured XPS spectrum. Notably, the absence of a C-Hf peak at 281.5 eV indicates that there is no carbon doping of the HfO_2 film. The presence of the C1s peak at 285 eV is due to adventitious carbon on the surface. The ratio of Hf:O is ~ 2:1 indicating a high quality, near stoichiometric, HfO_2 film.

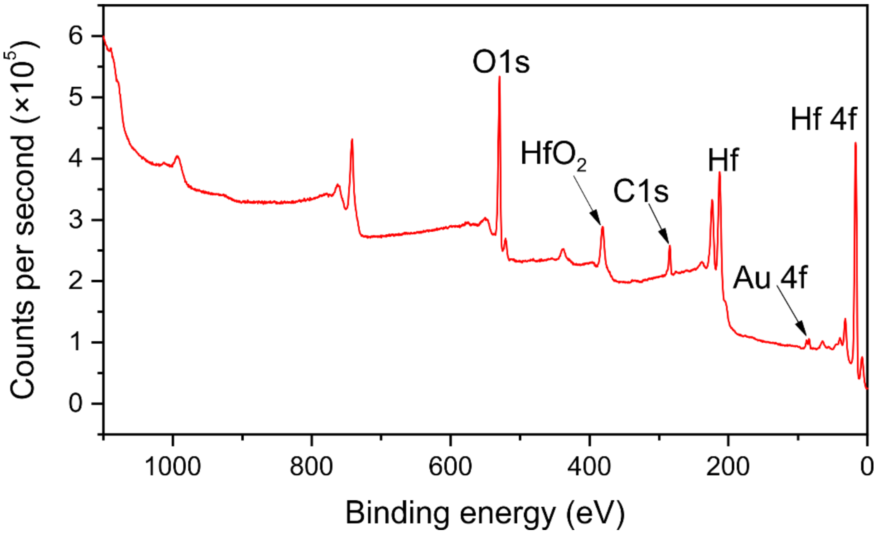

Fig. S4. X-ray photoemission spectrum of the HfO_2 layer.

Negative Differential Resistance

To confirm the hysteretic behavior and tunneling origin of the measured current, a thinner sample with a 3 nm thick HfO_2 layer was prepared. The I-V behavior displays (Fig S5) a similar hysteresis to the 5 nm PFTJ-RAM, as depicted in Fig. 2A. Notably, there is an order of magnitude increase in the measured current, confirming that the current is a tunneling current as opposed to a resistive conducting current such as that of a conducting filament type resistive RAM (34). The pronounced asymmetry of the tunnel current is attributed to higher contribution of Au-HfO_2 interfaces and the presence of an NDR region on the positive side of the I-V curve at V_b ~ 1.25 V. This behavior is due to the fact that E_i is higher for a thinner sample, thus, resulting in a greater opposition to the applied E_b. However, the NDR vanishes for V_b > 1.8 V since the traps are completely filled, and E_i no longer increases with V_b. Similar behavior has been observed in ZnO (35), $PbZrO_3$ (36), and $PbZr_{0.48}$ $Ti_{0.52}$ O_3 (37).

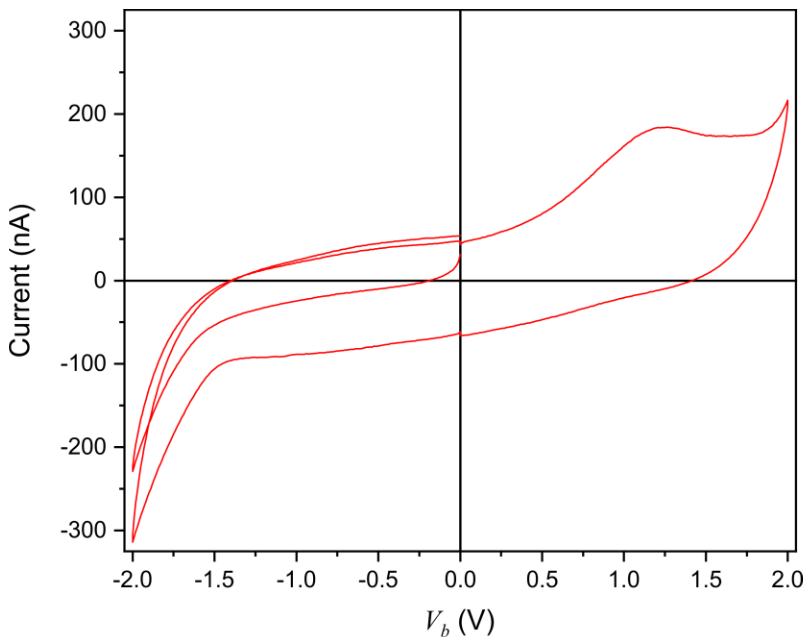

Fig. S5. Current-voltage curve of the PFTJ-RAM having a 3 nm thick HfO_2 layer. Evidence of negative differential resistance is present for V_b> +1.25V.

SPP mode profile

Fig. S6 depicts the SPP mode profile of a single cell of the PFTJ-RAM device.

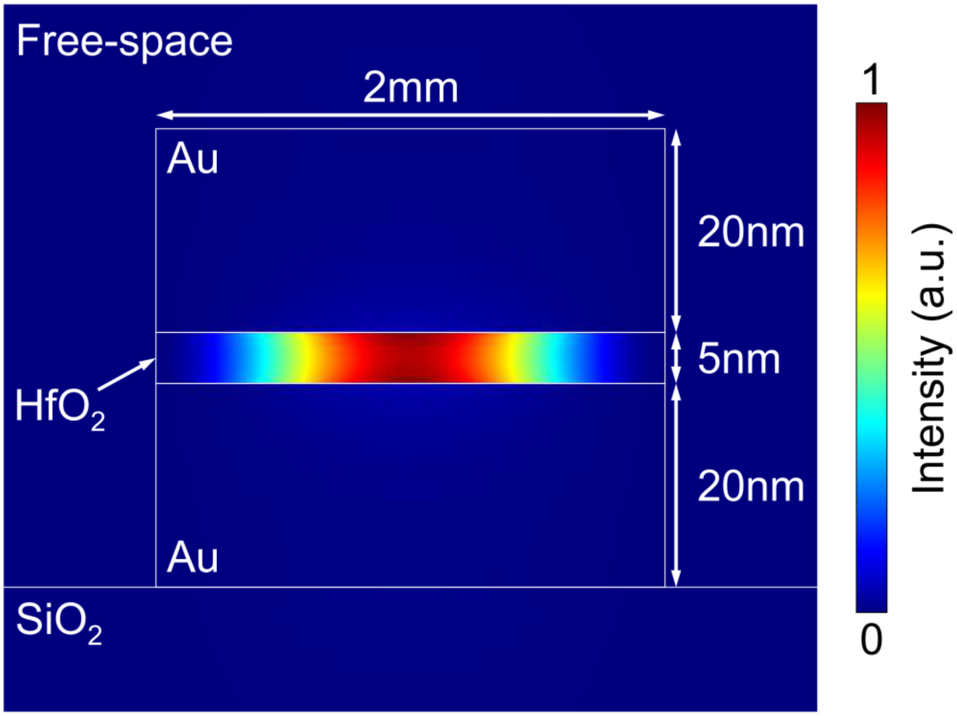

Fig. S6. **Plasmonic mode profile.** Plasmonic mode profile of single cell PFTJ-RAM consisting of a 5nm HfO_2 ferroelectric layer sandwiched between 20nm Au electrodes when excited by 800nm wavelength laser light.

SPP-induced tunneling current

Fig. S7 depicts a schematic of the illumination setup indicating direction of *s*- and *p*-polarization and SPP coupling angle, θ_{SPP}. Fig. S8 depicts the measured tunnel current induced by the SPP in a single PFTJ-RAM element for V_b = -0.5V to +0.5V in steps of 0.125V.

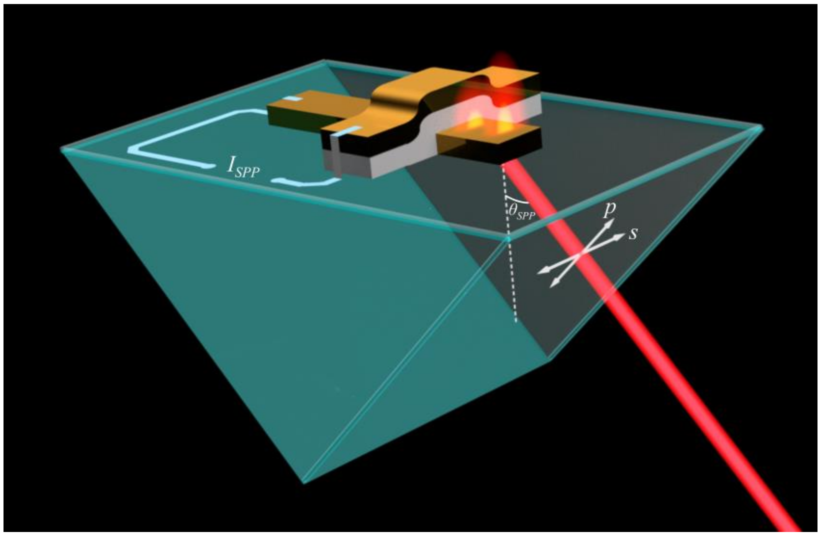

Fig. S7. Schematic depiction of the Kretschmann geometry used to facilitate SPP coupling for *p*-polarized laser light.

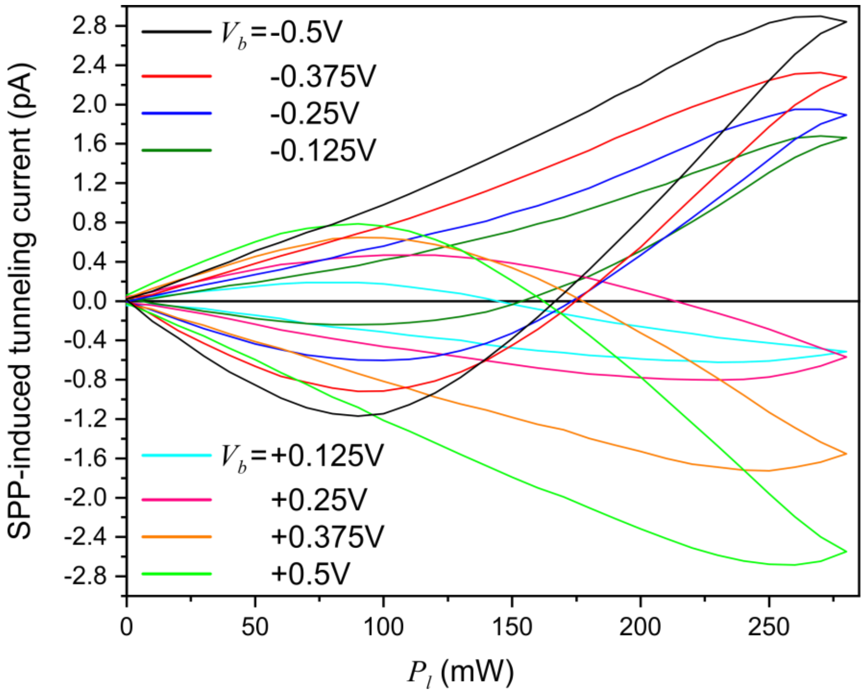

Fig. S8. SPP-induced tunneling current as a function of incident laser power for different bias voltages.

Absence of Plasmon Drag Effect

To determine the influence of the plasmon-drag (38, 39) effect on the measured current, we conducted current measurements on a sample comprising a single 45 nm thick layer of Au. The sample was excited by a SPP originating from both the right and left sides of the Au film and current was measured using a Keithley 619 Electrometer/Multimeter set at a measurement range of nA with 100 fA sensitivity. Fig. S9 illustrates the experimental results, which unequivocally demonstrate the absence of plasmon-drag generated current.

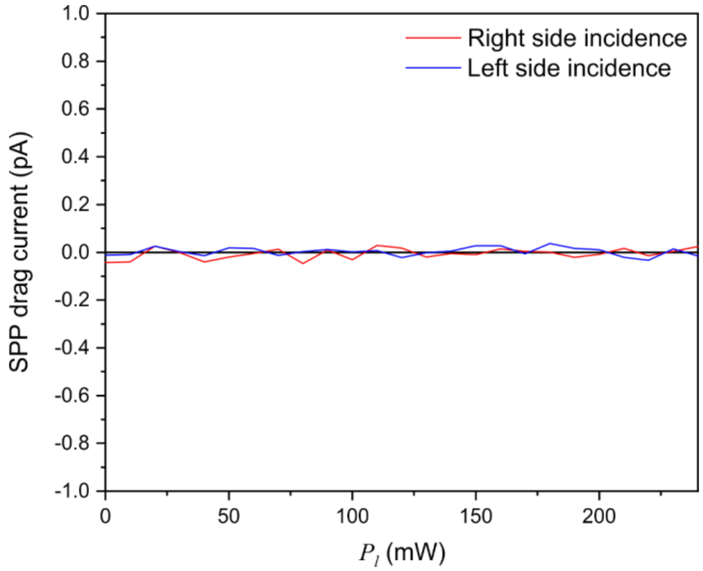

Fig. S9. **Absence of plasmon-drag effect when the SPP is excited from either the right or left side of a single 45 nm thick Au film.**

Ferroelectric behavior and depolarization field in amorphous HfO_2

The two polarization states in ferroelectrics arise due to the displacement of positively charged metallic ions from negatively charged oxygen ions upon application of an external electric field. In order to switch the polarization state, the applied electric field must be above the coercive field, E_c, which results in the typical hysteresis loop of the polarization vs. voltage curve of a FE material. This switching of polarization states has led to widespread use of FE materials in non-volatile RAM devices. However, it is well-known that HfO_2 films can exhibit ferroelectric (FE) behavior. The origin of the ferroelectricity is ascribed to the particular phase of the material (25, 26, 40). The monoclinic phase (*m*-HfO_2) does not exhibit ferroelectricity while the orthorhombic phase does. However, the *m*-HfO_2 phase is more prevalent than the orthorhombic phase. As such, there are a variety of methods used to induce ferroelectricity in *m*-HfO_2, including intentional doping with Si, Al, Y, Sr, La, and Gd (41) or unintentional doping with C, controlling the ozone dose during atomic layer deposition (ALD) (41), or the presence of sub-4 nm HfO_2 grains (42).

When a thin film of a ferroelectric material is sandwiched between metallic contacts, such as the PFTJ-RAM described here, the FE polarization, P, induces surface charge, σ_{pol}, at the ferroelectric-metal interface. The free charges in the metal will attempt to screen σ_{pol}, introducing screening charge of opposite sign, σ_s, at the ferroelectric interface of each electrode. This, in turn, will generate a space-charge region at each interface that are directed in the same direction, as depicted in Fig. S10. To preserve short-circuit boundary conditions of the structure, a depolarizing field, E_{depol}, must appear in the ferroelectric film. This depolarizing field is directed opposite to the

polarization and cancels out the field contribution from the dipoles at the interfaces. However, for thin ferroelectric films, due to the high value of the saturation polarization, σ_s cannot completely screen σ_{pol}, resulting in a higher E_{depol} within the FE material.

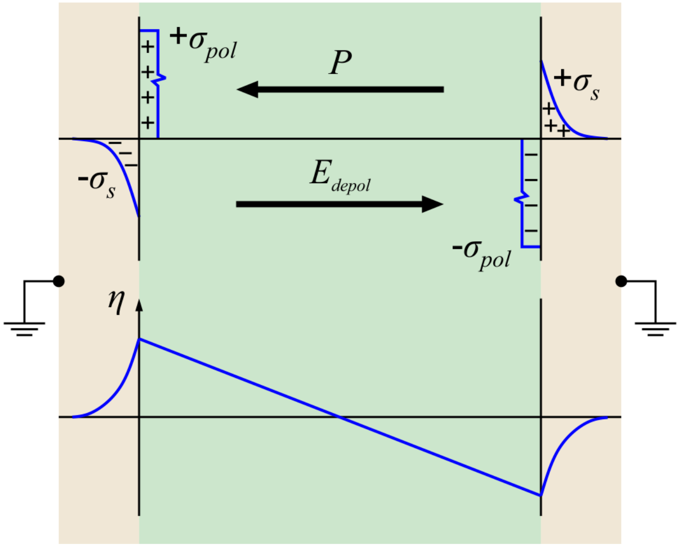

Fig. S10. **Diagram showing the depolarization field and the built-in electrostatic potential arising from incomplete polarization surface charge screening by the metallic electrodes.**

SPP-Induced Current

The PFTJ-RAM exhibits several potential sources of SPP-induced current, including hot electron tunneling between the metal layers and SPP excitation of oxygen vacancies within the HfO_2 layer. By analyzing the variation of measured current with a fixed P_l and changing V_b, we can determine the primary electron transport process. If SPP excites hot electrons within the metal film, these electrons will subsequently tunnel through the HfO_2 barrier. Consequently, the measured current will be influenced by both P_l (which determines the total number of excited hot electrons) and V_b (which modifies the height and shape of the HfO_2 tunnel barrier). On the other hand, electrons excited from oxygen vacancies at $\mathbf{V}^-$ and $\mathbf{V}^{2-}$ to the conduction band of HfO_2 by SPP will solely be influenced by P_l (which determines the total number of excited electrons), while V_b solely determines the direction of electron flow. Since the measured current is influenced by both V_b and P_l, it suggests that the dominant electron transport mechanism in the PFTJ-RAM is the tunneling of SPP-excited hot electrons.

REFERENCES AND NOTES

1. J. Boukhobza, P. Olivier, *Flash Memory Integration: Performance and Energy Issues* (Elsevier, ed. 1, 2017).

2. Y. Nishi, B. Magyari-Kope, Eds., *Advances in Nonvolatile Memory and Storage Technology* (Woodhead Publishing, 2019).

3. A. Chen, A review of emerging non-volatile memory (NVM) technologies and applications. *Solid State Electron.* **125**, 25–38 (2016).

4. H. Ishiwara, M. Okuyama, Y. Arimoto, Eds., *Ferroelectric Random Access Memories: Fundamentals and Applications* (Springer, 2004).

5. C. Weilenmann, A. N. Ziogas, T. Zellweger, K. Portner, M. Mladenović, M. Kaniselvan, T. Moraitis, M. Luisier, A. Emboras, Single neuromorphic memristor closely emulates multiple synaptic mechanisms for energy efficient neural networks. *Nat. Commun.* **15**, 6898 (2024).

6. F. Wang, Y. Liu, T. X. Hoang, H. S. Chu, S. J. Chua, C. A. Nijhuis, CMOS-compatible electronic–plasmonic transducers based on plasmonic tunnel junctions and Schottky diodes. *Small* **18**, e2105684 (2022).

7. U. Koch, C. Uhl, H. Hettrich, Y. Fedoryshyn, D. Moor, M. Baumann, C. Hoessbacher, W. Heni, B. Baeuerle, B. I. Bitachon, Plasmonics—High-speed photonics for co-integration with electronics. *Jpn. J. Appl. Phys.* **60**, SB0806 (2021).

8. M. I. Stockman, K. Kneipp, S. I. Bozhevolnyi, S. Saha, A. Dutta, J. Ndukaife, N. Kinsey, H. Reddy, U. Guler, V. M. Shalaev, Roadmap on plasmonics. *J. Opt.* **20**, 043001 (2018).

9. B. J. Shastri, C. Huang, A. N. Tait, P. R. Prucnal, Photonics for artificial intelligence and neuromorphic computing. *Nat. Photonics* **15**, 102–114 (2021).

10. T. J. Davis, D. E. Gómez, A. Roberts, Plasmonic circuits for manipulating optical information. *Nanophotonics* **6**, 543–559 (2017).

11. E. Ozbay, Plasmonics: Merging photonics and electronics at nanoscale dimensions. *Science* **311**, 189–193 (2006).

12. A. Emboras, I. Goykhman, B. Desiatov, N. Mazurski, L. Stern, J. Shappir, U. Levy, Nanoscale plasmonic memristor with optical readout functionality. *Nano Lett.* **13**, 6151–6155 (2013).

13. D. Y. Lei, K. Appavoo, F. Ligmajer, Y. Sonnefraud, R. F. Haglund Jr., S. A. Maier, Optically triggered nanoscale memory effect in a hybrid plasmonic-phase changing nanostructure. *ACS Photonics* **2**, 1306–1313 (2015).

14. E. Gemo, S. G. C. Carrillo, C. R. De Galarreta, A. Baldycheva, H. Hayat, N. Youngblood, H. Bhaskaran, W. H. P. Pernice, C. D. Wright, Plasmonically-enhanced all-optical integrated phase-change memory. *Opt. Express* **27**, 24724–24737 (2019).

15. J. Liao, S. Dai, R. C. Peng, J. Yang, B. Zeng, M. Liao, Y. Zhou, HfO_2-based ferroelectric thin film and memory device applications in the post-Moore era: A review. *Fundam. Res.* **3**, 332–345 (2023).

16. J. Y. Park, D. H. Choe, D. H. Lee, G. T. Yu, K. Yang, S. H. Kim, G. H. Park, S. G. Nam, H. J. Lee, S. Jo, B. J. Kuh, D. Ha, Y. S. Kim, J. Heo, M. H. Park, Revival of ferroelectric memories based on emerging fluorite-structured ferroelectrics. *Adv. Mater.* **35**, e2204904 (2023).

17. M. H. Park, Y. H. Lee, T. Mikolajick, U. Schroeder, C. S. Hwang, Review and perspective on ferroelectric HfO_2-based thin films for memory applications. *MRS Commun.* **8**, 795–808 (2018).

18. P. Neutens, P. Van Dorpe, I. De Vlaminck, L. Lagae, G. Borghs, Electrical detection of confined gap plasmons in metal–insulator–metal waveguides. *Nat. Photonics* **3**, 283–286 (2009).

19. H. Choo, M. K. Kim, M. Staffaroni, T. J. Seok, J. Bokor, S. Cabrini, P. J. Schuck, M. C. Wu, E. Yablonovitch, Nanofocusing in a metal–insulator–metal gap plasmon waveguide with a three-dimensional linear taper. *Nat. Photonics* **6**, 838–844 (2012).

20. Z. Han, A. Y. Elezzabi, V. Van, Experimental realization of subwavelength plasmonic slot waveguides on a silicon platform. *Opt. Lett.* **35**, 502–504 (2010).

21. R. He, H. Wu, S. Liu, H. Liu, Z. Zhong, Ferroelectric structural transition in hafnium oxide induced by charged oxygen vacancies. *Phys. Rev. B* **104**, L180102 (2021).

22. T. S. Böscke, J. Müller, D. Bräuhaus, U. Schröder, U. Böttger, Ferroelectricity in hafnium oxide thin films. *Appl. Phys. Lett.* **99**, (2011).

23. K. D. Kim, M. H. Park, H. J. Kim, Y. J. Kim, T. Moon, Y. H. Lee, S. D. Hyun, T. Gwon, C. S. Hwang, Ferroelectricity in Undoped-HfO_2 thin films induced by deposition temperature control during atomic layer deposition. *J. Mater. Chem. C* **4**, 6864–6872 (2016).

24. M. Pešić, F. P. G. Fengler, L. Larcher, A. Padovani, T. Schenk, E. D. Grimley, X. Sang, J. M. LeBeau, S. Slesazeck, U. Schroeder, T. Mikolajick, Physical mechanisms behind the field-cycling behavior of HfO_2-based ferroelectric capacitors. *Adv. Funct. Mater.* **26**, 4601–4612 (2016).

25. F. Yan, Y. Wu, Y. Liu, P. Ai, S. Liu, S. Deng, K. H. Xue, Q. Fuad, W. Dong, Recent progress on defect-engineering ferroelectric HfO_2: The next step forward via multiscale structural optimization. *Mater. Horiz.* **11**, 626–645 (2024).

26. E. D. Grimley, T. Schenk, X. Sang, M. Pešić, U. Schroeder, T. Mikolajick, J. M. LeBeau, Structural changes underlying field-cycling phenomena in ferroelectric HfO_2 thin films. *Adv. Electron. Mater.* **2**, 1600173 (2016).

27. A. Pal, V. K. Narasimhan, S. Weeks, K. Littau, D. Pramanik, T. Chiang, Enhancing ferroelectricity in dopant-free hafnium oxide. *Appl. Phys. Lett.* **110**, 022903 (2017).

28. C. Y. Huang, C. Y. Huang, T. L. Tsai, C. A. Lin, T. Y. Tseng, Switching mechanism of double forming process phenomenon in ZrO_x/ HfO_y bilayer resistive switching memory structure with large endurance. *Appl. Phys. Lett.* **104**, 062901 (2014).

29. R. R. Mehta, B. D. Silverman, J. T. Jacobs, Depolarization fields in thin ferroelectric films.*J. Appl. Phys.* **44**, 3379–3385 (1973).

30. M. Y. Zhuravlev, R. F. Sabirianov, S. S. Jaswal, E. Y. Tsymbal, Giant electroresistance in ferroelectric tunnel junctions. *Phys. Rev. Lett.* **94**, 246802 (2005).

31. C. Clavero, Plasmon-induced hot-electron generation at nanoparticle/metal-oxide interfaces for photovoltaic and photocatalytic devices. *Nat. Photonics* **8**, 95–103 (2014).

32. J. L. Gavartin, D. Muñoz Ramo, A. L. Shluger, G. Bersuker, B. H. Lee, Negative oxygen vacancies in HfO_2 as charge traps in high-*k* stacks. *Appl. Phys. Lett.* **89**, 082908 (2006).

33. T. Muneshwar, K. Cadien, Probing initial-stages of ALD growth with dynamic in situ spectroscopic ellipsometry. *Appl. Surf. Sci.* **328**, 344–348 (2015).

34. T. C. Chang, K. C. Chang, T. M. Tsai, T. J. Chu, S. M. Sze, Resistance random access memory. *Mater. Today* **19**, 254–264 (2016).

35. M. S. Kadhim, F. Yang, B. Sun, Y. Wang, T. Guo, Y. Jia, L. Yuan, Y. Yu, Y. Zhao, A resistive switching memory device with a negative differential resistance at room temperature. *Appl. Phys. Lett.* **113**, 053502 (2018).

36. E. M. Alkoy, T. Shiosaki, Electrical properties and leakage current behavior of un-doped and Ti-doped lead zirconate thin films synthesized by sol–gel method. *Thin Solid Films* **516**, 4002–4010 (2008).

37. Y. Podgorny, K. Vorotilov, A. Sigov, Negative differential conductivity in thin ferroelectric films. *Appl. Phys. Lett.* **105**, 182904 (2014).

38. M. Durach, A. Rusina, M. I. Stockman, Giant surface-plasmon-induced drag effect in metal nanowires. *Phys. Rev. Lett.* **103**, 186801 (2009).

39. T. Ronurpraful, D. Keene, N. Noginova, Plasmon drag effect with sharp polarity switching. *New J. Phys.* **22**, 043002 (2020).

40. M. H. Park, T. Schenk, C. M. Fancher, E. D. Grimley, C. Zhou, C. Richter, J. M. LeBeau, J. L. Jones, T. Mikolajick, U. Schroeder, A Comprehensive study on the structural evolution of HfO_2 thin films doped with various dopants. *J. Mater. Chem. C* **5**, 4677–4690 (2017).

41. M. H. Park, Y. H. Lee, H. J. Kim, Y. J. Kim, T. Moon, K. D. Kim, J. Müller, A. Kersch, U. Schroeder, T. Mikolajick, C. S. Hwang, Ferroelectricity and antiferroelectricity of doped thin HfO_2-based films. *Adv. Mater.* **27**, 1811–1831 (2015).

42. R. Materlik, C. Künneth, A. Kersch, The origin of ferroelectricity in $Hf_{1-x}Zr_xO_2$: A computational investigation and a surface energy model. *J. Appl. Phys.* **117**, 134109 (2015).
